# An Investigation of the Complexities of Successful and Unsuccessful Guide Dog Matching and Partnerships

**DOI:** 10.3389/fvets.2016.00114

**Published:** 2016-12-16

**Authors:** Janice Lloyd, Claire Budge, Steve La Grow, Kevin Stafford

**Affiliations:** ^1^Discipline of Veterinary Sciences, James Cook University, Townsville, QLD, Australia; ^2^College of Health, Massey University, Palmerston North, New Zealand; ^3^Institute of Veterinary, Animal and Biomedical Sciences, Massey University, Palmerston North, New Zealand

**Keywords:** guide dogs, matching success, human–animal relationships, vision impairment, blind mobility

## Abstract

Matching a person who is blind or visually impaired with a guide dog is a process of finding the most suitable guide dog available for that individual. Not all guide dog partnerships are successful, and the consequences of an unsuccessful partnership may result in reduced mobility and quality of life for the handler (owner), and are costly in time and resources for guide dog training establishments. This study examined 50 peoples’ partnerships with one or more dogs (118 pairings) to ascertain the outcome of the relationship. Forty-three of the 118 dogs were returned to the guide dog training establishment before reaching retirement age, with the majority (*n* = 40) being categorized as having dog-related issues. Most (*n* = 26) of these dogs’ issues were classified as being behavioral in character, including work-related and non-work-related behavior, and 14 were due to physical causes (mainly poor health). Three dogs were returned due to matters relating to the handlers’ behavior. More second dogs were returned than the handlers’ first or third dogs, and dogs that had been previously used as a guide could be rematched successfully. Defining matching success is not clear-cut. Not all dogs that were returned were considered by their handlers to have been mismatched, and not all dogs retained until retirement were thought to have been good matches, suggesting that some handlers were retaining what they considered to be a poorly matched dog. Almost all the handlers who regarded a dog as being mismatched conceded that some aspects of the match were good. For example, a dog deemed mismatched for poor working behavior may have shown good home and/or other social behaviors. The same principle was true for successful matches, where few handlers claimed to have had a perfect dog. It is hoped that these results may help the guide dog industry identify important aspects of the matching process, and/or be used to identify areas where a matching problem exists.

## Introduction

The guide dog, like the long cane, is a primary mobility aid intended to enhance the lifestyle of people with a visual disability (blind or visually impaired) by facilitating independent travel (1–5). Additional benefits imparted to a guide dog handler (the person who uses a guide dog) include friendship, companionship, increased social function, and improved self-esteem and confidence (3, 4, 6–14).

The process of producing guide dogs involves the selection and breeding of suitable dogs, raising and socialization of the pups, and their subsequent training as mobility aids (15–19). The making of a handler-guide dog pairing involves the matching of a trained dog to its handler, the training of the handler and dog as a team, and ongoing follow-up. Matching a person who has a visual disability with a guide dog is a process of finding the most suitable guide dog available for that individual, and a successful match is one of ongoing satisfaction with the partnership (20). However, not all guide dog partnerships are successful, and the consequences of an unsuccessful partnership may be severe in terms of the reduction in mobility and quality of life for the handler, and time and resources for guide dog training establishments (3, 4, 21, 22).

Guide dog schools worldwide pay a great deal of attention to the process of matching a dog to its new handler, but little evaluation has been carried out regarding the matching process or its subsequent outcome. Although a guide dog is principally an aid to mobility, the success of a team is not solely dependent on the dog’s ability to lead an individual safely and efficiently through the environment (23, 24). Factors other than orientation and mobility (O&M) such as those relating to social situations and home environment are considered when making matches (25). Lane et al. (26) suggested that the dog’s effects on enhancing the handler’s social interactions may be at least as important as increased mobility and independence. This suggestion was supported in a study of first time guide dog handlers (24) who found that the dog’s effects on the handler’s social interactions appeared to be a significant predictor of matching success. Matching is an art as much as a science, and there may be no such thing as a perfect match. Hence, the purpose of this research was to explore handler and guide dog relationships, from the handlers’ perspective, to identify characteristics of handler and dog that influence the success or failure of the match.

## Materials and Methods

This study examined 50 peoples’ (26 females and 24 males) partnerships with one or more dogs (118 pairings). All the dogs in the study were trained by guide dog schools that are members of the International Guide Dog Federation (IGDF), and as such are accredited to the highest international standards. The method of participant recruitment is described in Lloyd et al. (3) (p. 21). Descriptive and inferential statistical techniques were used to analyze the data.

The study was carried out in accordance with the recommendations of Massey University Human Ethics Committee with written informed consent from all subjects.

### Sample Description

To differentiate between the human and canine elements in the study, the term “handler” or “dog” will be used when referring to the 118 handler-dog teams (pairings), and the term “participant” will be used when referring in general to the 50 individuals involved in the study.

At the time the study was conducted, 39 of the 50 participants were currently using a dog; 14 were currently using their first dogs, 13 their second, 7 their third, 2 their fourth, 1 person a sixth, and 2 people were using their eighth dog. At this time, the age of the participants ranged from 21 to 86 years, with a mean of 50.3 years (SD = 15.61). Participants were on average 37.6 years old (SD = 15.46) when they received their first guide dog, with an age range from 17 to 75 years. More than a fifth of participants (*n* = 11) were not currently using a dog. Eight participants had decided not to use a dog in the future due to: having a limited workload (*n* = 3); family pressure/unsuitable living environment (*n* = 3); and two people whose relationship with the guide dog school had foundered. The remaining three participants were on the waiting list for a replacement dog. Information on how the end of the relationship affects people’s desire to apply for a replacement can be found in Lloyd et al. (21).

Of the 118 dogs in the sample, 66.9% (*n* = 79) had been retired or withdrawn before the study commenced and 33.1% (*n* = 39) were currently in work (Table 1). There were nearly twice as many bitches as male dogs in the sample, and both sexes were neutered except for one male.^1^ The Labrador Retriever was the most commonly used breed (57.6%), 11% were German Shepherd dogs, 11% were Labrador/Golden Retrievers (first crosses), and 4.3% were Golden Retrievers. Other breeds, including mix-breeds and “exotics” like Standard Poodles, Boxers, Giant Schnauzers, and Flat and Curly Coated Retrievers comprised 16.1% of the sample. Coat color was predominately yellow (39%) or black (36.5%) (Table 1).

**Table 1:** **Canine (*N* = 118) demographic data**.

Canine demographic data	Dog 1(*n* = 50)	Dog 2(*n* = 32)	Dog 3(*n* = 15)	Dog 4(*n* = 8)	Dog 5(*n* = 5)	Dog 6(*n* = 4)	Dog 7(*n* = 2)	Dog 8(*n* = 2)	O/all(*N* = 118)
All dogs (*n*)	50	32	15	8	5	4	2	2	118
Months worked—range	1–138	1–144	4–132	2–156	3–96	2–24	9–24	42–72	1–156
Months worked (M)	70.22	46.56	48.33	39.63	37.80	12.00	16.50	57.00	54.47
Months worked (SD)	41.30	41.53	44.73	57.56	40.49	11.66	10.61	21.21	43.76

Current dogs (*n*)	14	13	7	2	0	1	0	2	39
Months worked—range	14–132	9–120	4–106	2–26	N/a	N/a	N/a	42–72	2–132
Months worked (M)	71.50	50.23	28.43	14.00	N/a	20	N/a	57.00	51.67
Months worked (SD)	42.98	32.90	35.76	16.97	N/a	N/a	N/a	21.21	39.28

Previous dogs (*n*)	36	19	8	6	5	3	2	0	79
Months worked—range	1–138	1–144	6–132	3–156	3–96	2–24	9–24	N/a	1–156
Months worked (M)	69.72	44.05	65.75	48.17	37.80	9.33	16.50	N/a	55.85
Months worked (SD)	41.24	47.25	46.51	65.05	40.49	12.70	10.61	N/a	45.98

**All dogs—breed (%)**									
Labrador Retriever	62.0	59.4	40.0	62.5	80.0	50.0	50.0	0	57.6
Golden Retriever	4.0	6.3	6.7	0	0	0	0	0	4.3
Lab Ret. × Golden Ret.	14.0	6.3	20.0	12.5	0	0	0	0	11.0
German Shepherd dog	12.0	12.5	13.3	0	0	0	0	50.0	11.0
Exotic/others	8.0	15.6	20.0	25.0	20.0	50.0	50.0	50.0	16.1

**All dogs—sex (%)**									
Male castrate^a^	34.0	43.7^a^	33.3	37.5	60.0	25.0	0	50.0	37.3^a^
Female spayed	66.0	56.3	66.7	62.5	40.0	75.0	100	50.0	62.7

**All dogs—color (%)**									
Yellow	46.0	46.9	26.7	12.5	20.0	50.0	0	0	39.0
Black	38.0	28.1	46.7	50.0	80.0	0	0	0	36.5
Chocolate	2.0	6.3	6.7	12.5	0	25.0	50.0	0	5.9
Black and Tan	14.0	12.5	6.7	12.5	0	0	50.0	50.0	12.7
Others	0	6.2	13.3	12.5	0	25.0	0	50.0	5.9

*^a^One dog not neutered (0.9%)*.

*N/a, not applicable*.

#### Independence of Errors

Most of the participants (*n* = 32) had used more than one dog and were serially represented in the database. Hence, an “intra-class correlation coefficient” (ICC) was calculated to test for any “non-independent” observations (caused by potential clustering) on the outcome of matching success^2^ using the values shown in Table 2 and the following formula provided by Snedechor and Cochran (27) (pp. 242–244):

$$\text{ICC}=\rho =\frac{\text{MSB}-\text{MSW}}{\text{MSB}+\text{MSW}(\bar{n}-1)}=-0.086$$

where *n*-bar = average group size = 118/50 = 2.36, MSB = mean square between subjects = 0.169, and MSW = mean square within subjects = 0.214.

**Table 2:** **Tests of between-subjects-effects generated to calculate the intra-class correlation coefficient for the outcome (dependent) variable of matching success**.

Source	Type 111 sum of squares	df	Mean square	*F*	Sig. *p*
Corrected model	8.281^a^	49	0.169	0.788	0.808
Intercept	4.828	1	4.828	22.525	0.000
All cases	8.281	49	0.169	0.788	0.808
Error	14.575	68	0.214	–	–
Total	31.000	118	–	–	–
Corrected total	22.856	117	–	–	–

*^a^R^2^ = 0.362; adjusted R^2^ = 0.097*.

The resulting value (ICC = −0.086) was very small and negative, which according to Snedechor and Cochran (27) argues strongly against there being any meaningful positive correlation between measurements within the same handler. This value, along with the average number of dogs used in the sample being only 2.4 (118 dogs/50 people), supported the decision not to make any adjustments and to treat each handler-dog pairing as an independent observation.

### Data Collection

Data was collected *via* a structured self-report questionnaire (Data Sheet S1 in Supplementary Material) that was delivered *via* telephone to 39 participants and face-to-face for 11. The method of data collection was either chosen by the participant or was by way of necessity due to the logistics of travel. There did not appear to be any discernible difference in the quality of the data obtained by the two methods of data collection.

Demographic data (e.g., age, gender) was collected on each participant. Participants were asked to comment on the “good” and “bad” behavioral and physical characteristics of each dog they had used and to rate the importance of these traits. Participants were also asked about the outcome of the handler-dog partnership regarding whether the dog was currently working, retired,^3^ accidentally deceased, or had been returned^4^ to the guide dog school. Further questions concerned: why dogs ceased working; mismatched dogs versus returned dogs; and reasons for successful and unsuccessful matches. As over a third (*n* = 17) of participants had used or were using dogs that had been matched with at least one other handler, participants were also asked to comment on the use of “rematched” dogs.

## Results

### Characteristics of “Good” and “Bad” Dogs

The handlers’ comments on what was good and bad, behaviorally and physically, about their dogs (118 pairings) are shown in decreasing order of frequency in Table 3. Handlers also rated the three most important traits within each of the four categories, denoted in Table 3 with one, two, or three asterisks in decreasing order of importance.

**Table 3:** **Good and bad canine (*N* = 118) behavioral and physical characteristics**.

Good traits	%	Bad traits	%
Behavioral		Behavioral	
Social inc., home; personality^a^	83.3	Specific guiding tasks	29.8
Work rate/capacity to work^b^	75.7	Distractions when working (mostly to dogs)^a^	28.1
Specific guiding tasks^c^	70.6	Work rate/capacity to work	27.2
Speed—control/tension/sustainability	28.9	Social inc., home; personality	27.2
Coping	23.0	Scavenging/food oriented salivation^b^	24.7
Not overly sensitive	17.9	Escapism/poor recall^c^	23.8
Office behavior	13.6	Aggression/s (mostly to dogs)	23.0
Toileting habits	11.1	Speed—control/tension/sustainability	22.1
No scavenging/food-oriented salivation	11.1	No bad	17.9
Good with children	10.2	Coping^c^	16.2
No escapism/good recall	9.4	Toileting habits	14.5
Good with other pets	6.0	Overly sensitive	14.5
Acceptable distractions	5.1	Office behavior	6.8
Barking only when appropriate	4.3	Coprophagous	5.1
No good	3.4	Chased cats	5.1
No aggressive tendencies	2.6	Suspicious—people/objects	4.3
Discouraged unwanted cats at home	1.7	Aggressive to other pets	3.4
Retrieved objects	0.9	Barking	3.4
Not coprophagous	0.9	Will not retrieve objects	0.9
		Fussy/expensive eating habits	0.9
		Anxious re-car travel	0.9

**Physical**		**Physical**	
Size (mostly compact)^a^	55.3	No bad	36.6
Breed^b^	48.5	Health^a^	27.2
Good-looking^c^	40.8	Coat—shedding/high maintenance^b^	22.1
Sex (mostly female)	32.3	Size (mostly too big)^c^	16.2
Easy-care coat (mostly short hair)	25.5	Strength—pulling	14.5
Color—compliment/unlike previous dog	23.0	Breed	12.8
Tactility—soft coat/ears	12.8	Gait—hard to follow/unstable/veering	4.3
Gait—easy to follow/provide stability	7.7	Not good-looking	4.3
Strength—pulling uphill only	6.8	Sex (mostly male)	2.6
Nothing remarkable/acceptable	6.0	Age—too puppy-like/too mature	2.6
Weight	4.3	Tail not docked—nuisance factor	2.6
Docked tail—no nuisance factor	1.7	Overweight	2.6
		Color—hair noticeable on clothing/carpets	1.7
		Not tactile—coarse texture of coat/ears	1.7
		Malodorous	1.7

*^a^Item most often cited as greatest importance for that category*.

*^b^Second most often cited*.

*^c^Third most often cited*.

*D, dog; H, handler; B, behavioral; P, physical; W, work; NW, non-work; N/a, not applicable*.

The most commonly mentioned good behavior was related to “social behavior” (83.3%) including the dog being personable and well behaved at home and in other social settings, followed by “work rate” (capacity/ability to work) (75.7%) and “specific guiding tasks” (70.6%). These three characteristics were also considered to be the most important. The bad behavior most commonly reported concerned “specific guiding tasks” (29.8%) closely followed by “distractions (mainly to other dogs) while working” (28.1%) and “work rate” (27.2%). The three traits most often cited as being of first, second, and third equal importance regarding undesirable behavior are “distractions (mainly to other dogs) while working,” “scavenging,” and poor “coping skills” or “running away,” respectively (Table 3).

Concerning physical characteristics, the “size” of the dog matters. Over half of the handlers (55.3% of 118 pairings) mentioned that they liked the size of their dogs, with most handlers preferring a compact dog as opposed to a large one—mainly for ease of fitting into confined spaces such as under a desk at work or in a transport vehicle. Tall handlers said that they required a dog to be big enough for them to use the harness handle effectively, but not to be taller or longer than necessary. Smaller dog were deemed easier to lift, bathe, and be less strong and hence not be able to pull as hard as a more powerfully built dog. “Breed” was the next frequently mentioned desirable physical trait (48.5%) followed by “attractiveness” (40.8%). One handler, who returned her dog said “I thought that everything would be alright if only my next dog was a Poodle,” but then described the Standard Poodle as being “dizzy” and “unfocussed” when it was received. Reasons given for wanting a dog to be attractive included “I feel like I live my life in a fish bowl, with everyone watching—so why shouldn’t my dog look nice?” and “I miss my old dog’s soft, soft ears. I don’t like [the new dog’s] ears. I don’t suppose [the instructor] would find that important as it’s not a mobility thing.” “Size,” “breed,” and “attractiveness” were also consecutively considered to be the three most important characteristics concerning physical traits (Table 3).

Over a third of handlers (36.6% of 118 pairings) stated that their dogs had no bad physical characteristics, 27.2% experienced dogs with health problems (including skin issues), and 22.1% expressed troublesome issues regarding the dogs’ coat such as shedding and amount of care required (grooming/bathing). “Health” was rated as being of most importance in this category, followed by “coat,” and the dog being too large in “size.”

#### Rating Canine Qualities

To gain a more specific understanding of what qualities were found attractive and unattractive in guide dogs, the 32 participants who had worked with more than one dog were asked to state the *main* characteristic that they liked best about their favorite dog (*n* = 32), and the *main* characteristic they liked least about their least favorite dog (*n* = 32). The 18 participants who used only one dog were asked to state the best and worst qualities of that particular dog (*n* = 18). Participants were asked to categorize these responses into either work (W) or non-work (NW) scenarios, and state if this was behavioral (B), physical (P), or emotional^5^ (E) in nature.

**Figure 1:**
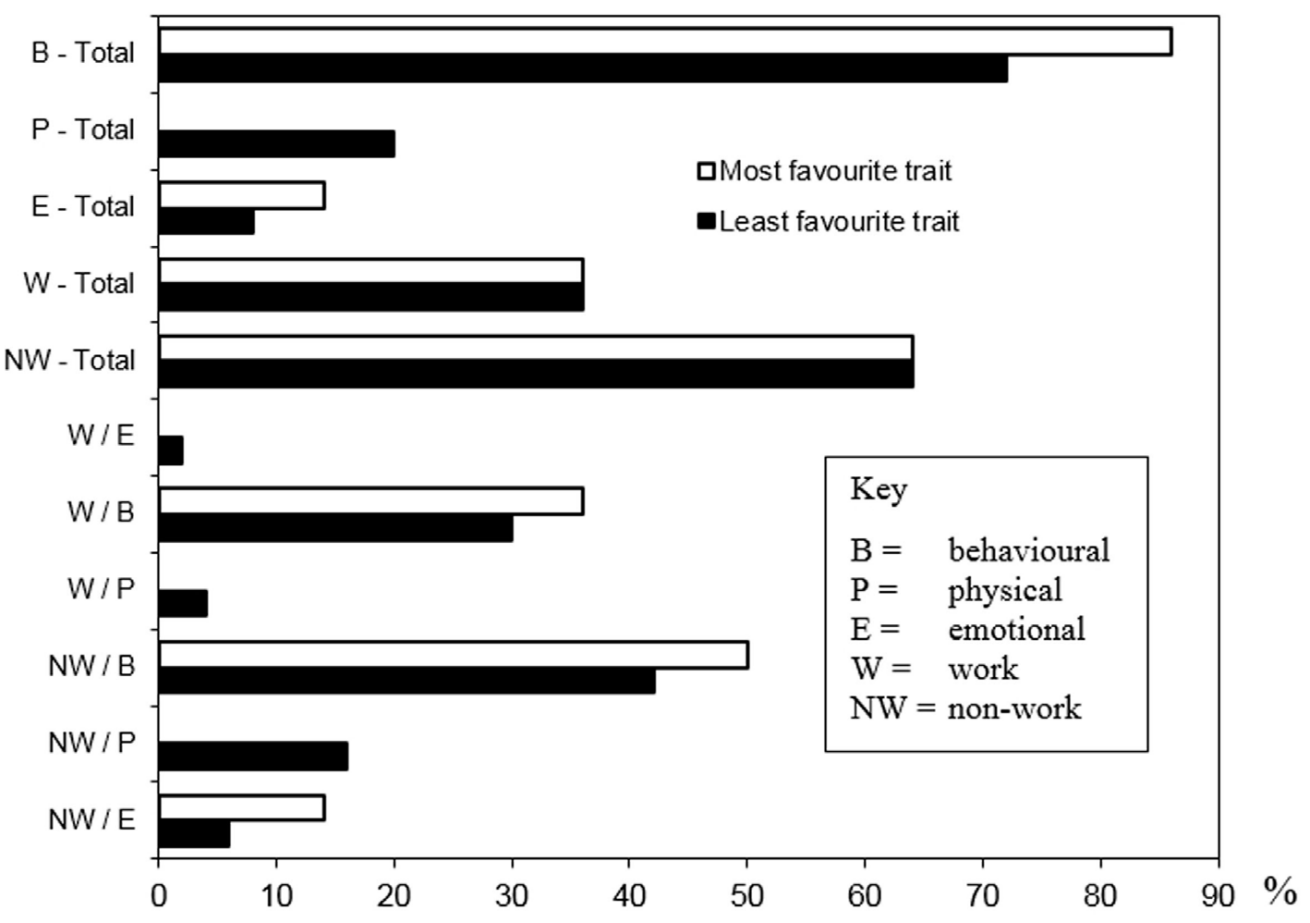
**The participants’ (*N* = 50) most favourite and least favourite characteristics of their dogs (*n* = 82) concerning behavior, physical, and emotional categories, and whether this relates to work or non-work**.

The results of these classifications (Figure 1) show that most of the favorite and least favorite traits were behavioral in nature. Specifically, half of the “favorite” responses were classified as non-working behavior (NW/B), followed by working behavior (W/B) (36.0%) and non-working emotional (NW/E) (14.0%). To illustrate with examples from this sample, the NW/B category is demonstrated by a dog that “was wonderful company at home”; W/B is shown by a dog that “was excellent at finding destinations”; and NW/E *via* a dog that “was a soulmate for almost 12 years.” Likewise, the majority of the “least favorite” responses were categorized as NW/B (42.0%). This was followed by W/B (30.0%), non-working physical (NW/P) (16.0%), NW/E (6.0%), working physical (W/P) (4.0%), and working emotional (W/E) (2.0%). Illustrations of NW/B included a dog that “solicited too much attention at social functions” and another that “growled at visitors at home.” A dog that “failed to stop consistently at the end of the pavement” (down kerb) exemplifies W/B, and a dog that “shed hair excessively” represents NW/P. The statement “I don’t know why I did not like that dog… it was quite a good worker and well behaved at home, but we just did not gel” was coded as NW/E. A dog that was categorized W/P was considered “too sick to work,” and a dog classified as W/E pertained to the inexperience of the handler who professed that he “did not know how to work with my first dog.”

Thirty of the 32 participants who had handled multiple dogs rated their dogs (*n* = 93) in order of favoritism. Sixteen of the 30 dogs considered to be the favorite had been the participants’ first guide dog, seven were subsequent dogs previously employed as a guide, and seven were dogs in current use.

### The Outcome of the Partnership

**Figure 2:**
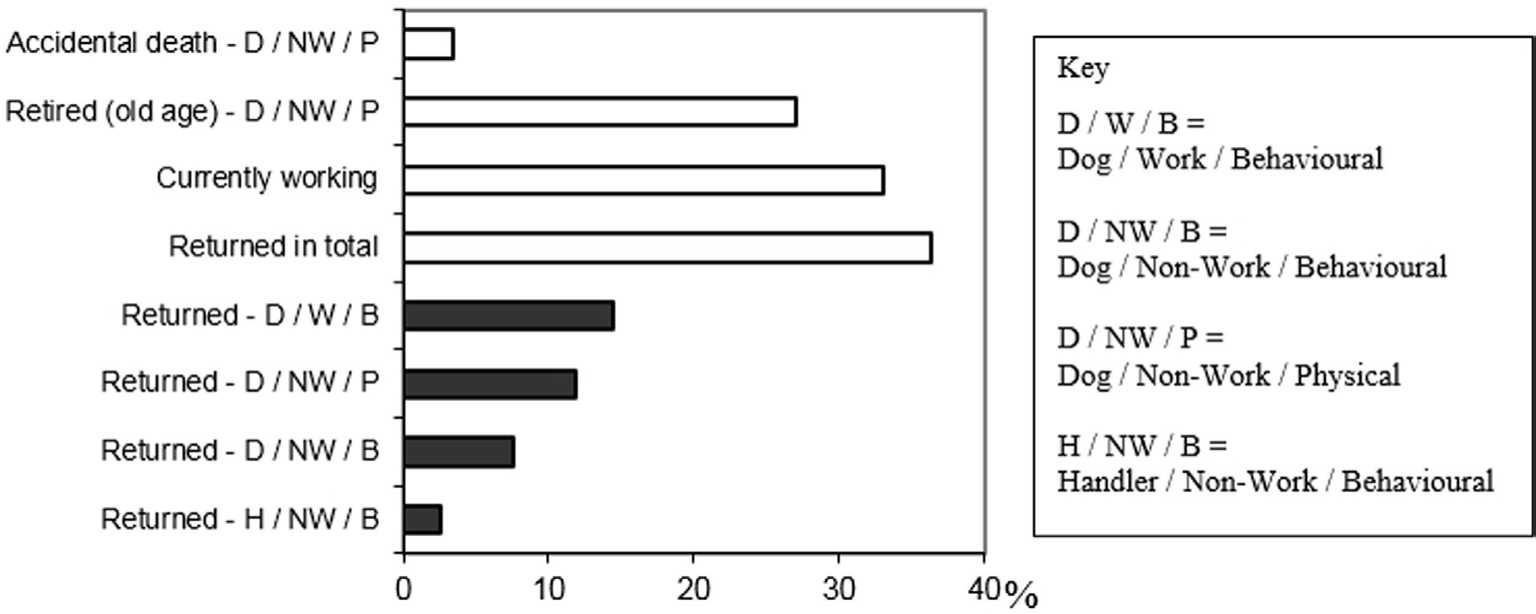
**The outcome of the dogs’ (*N* = 118) working lives**. The white bars show the broad outcomes and the black bars categorize the main reason why dogs were returned to the guide dog school.

The outcome of all the handler-dog partnerships (118 pairings) in terms of whether a dog was currently working, retired, accidentally deceased, or returned to the guide dog school is illustrated in Figure 2. One-third (33.1%) of dogs in the sample were currently working. Of the two-thirds (66.9%) that were not, 36.4% were returned to the guide dog school before the dog reached retirement age, 27.1% were retired due to disorders related to old age (poor health, failing eyesight, slowing down, etc.) and 3.4% died from accidental causes prior to retiring. An itemization of the dogs that were currently working and the main general and specific explanation for why dogs either were returned or were under consideration for return are shown in Table 4.

**Table 4:** **The outcome of the dogs’ (*N* = 118) working lives, and the general and specific categories for why dogs were returned or were being considered for return**.

Outcome status of dogs’ working lives	Dog 1(*n* = 50)	Dog 2(*n* = 32)	Dog 3(*n* = 15)	Dog 4(*n* = 8)	Dog 5(*n* = 5)	Dog 6(*n* = 4)	Dog 7(*n* = 2)	Dog 8(*n* = 2)	O/all(*N* = 118)
Currently working (%)	28.0	40.6	46.7	25.0	0	25.0	0	100	33.1
Retired—old age (≥8 years) (%)	40.0	18.8	26.7	25.0	0	0	0	0	27.1
Accidental death (≤8 years) (%)	4.0	0	6.7	0	0	0	50.0	0	3.4
Returned in total (≤8 years) (%)	28.0	40.6	20	50.0	100	75.0	50.0	0	36.4

**Returned—general (%)**									
Dog physical (D/P)	12.0	15.6	0	0	40.0	0	50.0	0	11.9
Dog behavior (D/B)	16.0	24.8	6.7	50.0	60.0	50.0	0	0	22.0
Handler behavior (H/B)	0	0	13.4	0	0	25.0	0	0	2.6

**Returned—D/P specific—health (%)**									
Musculoskeletal [non-work (NW)]	2.0	6.3	N/a	N/a	20.0	N/a	50.0	N/a	4.3
Cancer (malignant) (NW)	6.0	3.1	N/a	N/a	0	N/a	0	N/a	3.4
Endocrine (NW)	0	6.3	N/a	N/a	0	N/a	0	N/a	1.7
Gastrointestinal (NW)	2.0	0	N/a	N/a	0	N/a	0	N/a	0.9
Renal (NW)	2.0	0	N/a	N/a	0	N/a	0	N/a	0.9
Skin (NW)	0	0	N/a	N/a	20.0	N/a	0	N/a	0.9

**Returned—D/B specific (%)**									
Specific-guiding tasks (W)	2.0	0	0	0	20.0	0	0	0	1.7
Distracted/aggressive to dogs (W)	6.0	0	0	37.5	0	0	0	0	5.1
Social (inc., home) (NW)	4.0	12.5	6.7	0	20.0	25.0	0	0	7.6
Capacity to work (work rate) (W)	2.0	3.1	0	12.5	0	0	0	0	2.5
Coping (anxiety; adaptability) (W)	2.0	3.1	0	0	0	25.0	0	0	2.5
Working speed (W)	0	6.3	0	0	20.0	0	0	0	2.5

**Returned—H/B specific (%)**									
Temporary match (NW)	0	0	6.7	0	0	0	0	0	0.9
Family (NW)	0	0	6.7	0	0	0	0	0	0.9
Environment (NW)	0	0	0	0	0	25.0	0	0	0.9

**Currently working—general (%)**									
Going well	14.0	31.3	40.0	25.0	0	0	0	100	22.9
Good, but nearing retirement	12.0	3.1	0	0	0	0	0	0	5.9
Potential return—D/B and D/P	2.0	6.3	6.7	0	0	25.0	0	0	4.2

**Currently working, but being considered for return—specific (%)**									
Distract/aggress. To dogs (D/W/B)	2.0	0	0	0	0	0	0	0	0.9
Coping (D/W/B)	0	3.1	0	0	0	0	0	0	0.9
Scavenging (D/NW/B)	0	0	6.7	0	0	0	0	0	0.9
Aggressive to other pets (D/NW/B)	0	3.1	0	0	0	0	0	0	0.9
Ill-health—skin (D/NW/P)	0	0	0	0	0	25.0	0	0	0.9

#### Reasons Dogs Cease Working

Although the majority of dogs in the sample (66.9%) had ceased working, most (63.6%) had not been returned. The primary reasons for dogs not currently working or for being considered for return have been categorized as either dog (D) related or handler^6^ (H) related, grouped into work (W) versus NW scenarios, and considered behavioral (B) or physical (P) in nature. Results fell into four of the eight possible combinations, which are displayed in Figure 2.

Forty of the 43 dogs that were returned were returned for dog-related problems. Of these 40 dogs, most (*n* = 26) were returned for dog behavioral problems, with almost two-thirds (*n* = 17) related to working behavior and nine dogs for behaviors unrelated to work (i.e., poor social and/or home behaviors) (Table 4). Problems relating to working behavior included dogs being distracted by and/or aggressive to other dogs, coping skills, and specific-guiding tasks. The few handlers who returned their dogs for incompatible walking speed said it was “frightening and uncomfortable to be dragged around” by a dog going too fast, “frustrating to be held back” by a slow dog and “confusing” if speed was inconsistent as the handler may not know why the dog slowed. Fourteen dogs were returned due to physical causes, that is, health issues that were unrelated to work including musculoskeletal disorders and cancer. The remaining three dogs were returned for NW-related handlers’ behavior. One of these dogs had been matched on a temporary basis while the handler awaited a more permanent dog, one handler said he/she was pressurized not to have a dog in the workplace, and one handler’s partner did not want a dog living in the house.

Of the 39 dogs (33.1%) working at the time, the study was conducted, five were being considered for return because of various dog-related health and behavior problems. In general, more second dogs were returned than first or third dogs, respectively, and around twice the number of second and third dogs that were currently working were being considered for return compared with first.

Most of the handlers who regarded their dogs as mismatched conceded that some aspects of the match were good. For example, a dog deemed mismatched for poor work may have shown good home and/or other social behaviors. The same principle held true for successful matches, where few handlers claimed to have had a perfect dog. However, it is noteworthy that the majority of dogs (*n* = 24 of 26) returned primarily for D/B reasons exhibited more than one undesirable behavior. Eight of the nine dogs that were returned for D/NW/B (primarily for poor home and social behavior) also had behavioral problems related to work. These included low coping skills, poor work rate, easily distracted, overly sensitive, working speed too fast, and toileting on walks. Sixteen of the 17 dogs returned mainly for their W/B also displayed a range of NW-related problems including poor social behavior (*n* = 8) and three dogs were criticized for physical issues such as excessive hair shedding and for being a specific breed.

#### Classification of Outcome

The majority (73.7%, *n* = 87) of dogs in the sample were considered to have been successfully matched and 63.6% (*n* = 75) of all dogs were retained (Table 5). However, not all dogs that were returned before reaching retirement age were considered by their handlers to have been mismatched, and not all dogs retained until retirement were thought to have been good matches. Thus, the dogs were classified as:

Combination 1: successfully matched and retained (56.8%, *n* = 67)
Combination 2: mismatched, but retained (6.8%, *n* = 8)
Combination 3: successfully matched, but returned (17.0%, *n* = 20)
Combination 4: mismatched and returned (19.5%, *n* = 23).

**Table 5:** **Whether handlers deemed their dogs (*N* = 118) to be successfully matched or not and how this relates to the dogs being returned or retained**.

Dogs’ matching status and months worked	Dog 1(*n* = 50)	Dog 2(*n* = 32)	Dog 3(*n* = 15)	Dog 4(*n* = 8)	Dog 5(*n* = 5)	Dog 6(*n* = 4)	Dog 7(*n* = 2)	Dog 8(*n* = 2)	O/all(*N* = 118)
**Successfully matched in total (%)**	80.0	68.7	86.7	50.0	40.0	50.0	100	100	73.7
Months worked—range	1–132	1–144	4–132	2–156	62–96	2–20	9–24	42–72	1–156
Months worked—(M)	75.05	61.77	53.46	71.5	79	11	16.5	57	65.16
Months worked—(SD)	38.67	41.75	46.03	70.64	24.04	12.73	10.61	21.21	42.29

Mismatched in total (%)	20.0	31.3	13.3	50.0	60.0	50.0	0	0	26.3
Months worked—range	3–138	1–30	12–18	3–14	3–25	2–24	N/a	N/a	1–138
Months worked—(M)	50.9	13.1	15	7.75	10.33	13	N/a	N/a	24.45
Months worked—(SD)	47.86	8.01	4.24	5.62	12.7	15.56	N/a	N/a	32.81

Retained in total (%)	72.0	59.4	80.0	50.0	0	25.0	50.0	100	63.6
Months worked—range	12–138	9–144	4–132	2–156	N/a	N/a	N/a	42–72	2–156
Months worked—(M)	85.03	65.9	55.92	71.5	N/a	20	24	57	72.37
Months worked—(SD)	36.55	41.37	46.77	70.64	N/a	0	0	21.21	42.3

Returned in total (%)	28.0	40.6	20.0	50.0	100	75.0	50.0	0	36.4
Months worked—range	1–72	1–72	6–36	3–14	3–96	2–24	N/a	N/a	1–96
Months worked—(M)	32.14	18.31	18	7.75	37.8	9.33	9	N/a	23.23
Months worked—(SD)	25.76	20.76	15.88	5.62	40.49	12.7	0	N/a	24.68

**Combination 1**									
Successfully matched—retained (%)	62.0	53.1	73.3	50.0	0	25.0	50.0	100	56.8
Months worked—range	12–132	9–144	4–132	2–156	N/a	N/a	N/a	42–72	2–156
Months worked—(M)	85.71	70.94	59.36	71.5	N/a	20	24	57	74.03
Months worked—(SD)	35.78	40.78	47.42	70.64	N/a	0	0	21.21	41.87

**Combination 2**									
Mismatched—retained (%)	10.0	6.3	6.7	0	0	0	0	0	6.8
Months worked—range	14–138	16–30	N/a	N/a	N/a	N/a	N/a	N/a	14–138
Months worked—(M)	80.8	23	18	N/a	N/a	N/a	N/a	N/a	58.5
Months worked—(SD)	45.47	9.9	0	N/a	N/a	N/a	N/a	N/a	46.31

**Combination 3**									
Successfully matched—returned (%)	18.0	15.6	13.3	0	40.0	25.0	50.0	0	17.0
Months worked—range	1–72	1–72	6-36	N/a	62–96	N/a	N/a	N/a	1–96
Months worked—(M)	38.33	30.6	21	N/a	79	2	9	N/a	35.45
Months worked—(SD)	23.07	30.5	21.21	N/a	24.04	0	0	N/a	28.3

**Combination 4**									
Mismatched—returned (%)	10.0	25.0	6.7	50.0	60.0	50.0	0	0	19.5
Months worked—range	3–72	1–18	N/a	3–14	3–25	2-24	N/a	N/a	1–72
Months worked—(M)	21	10.63	12	7.75	10.33	13	N/a	N/a	12.61
Months worked—(SD)	29.16	5.78	0	5.62	12.7	15.56	N/a	N/a	14.76

##### Combination 1: Successfully Matched and Retained

Most of the dogs (56.8%, *n* = 67) in the sample were considered to be well matched and were kept by their handlers. Three dogs whose handlers believed them to be poor mobility aids were included in this group. This was due to the handlers feeling that they had enough useful residual vision to compensate for the dogs’ lack of abilities and/or because the dogs were considered good companions.

##### Combination 2: Mismatched but Retained

The 6.8% (*n* = 8) of dogs that were considered mismatched, but not returned, were retained for several reasons. These included three participants who had used more than one dog becoming emotionally attached to their first dogs and being highly motivated to make the partnership work, and another who claimed not to have known any better as it was his first dog and he had nothing to compare it to. The other four dogs that were retained despite being unsuccessfully matched were four of the five dogs in current use that were being considered for return (Table 4) for reasons of: being distracted by and aggressive to other dogs when working; being aggressive to other pets; being overly sensitive and not coping with the demands of guiding; and scavenging on and off the job.

##### Combination 3: Successfully Matched but Returned

Twenty dogs in the sample (17.0%) were returned before the dogs reached retirement age despite being successfully matched. Of these dogs, 12 were returned due to the dogs’ unexpected ill-health, one dog was returned for slowing down through the normal aging process as it neared retirement age, one dog was withdrawn by the guide dog school because of protective aggressive tendencies, one dog was returned as it had been matched on a temporary basis until the handler’s preference for a younger dog could be fulfilled, one dog was swapped with a close associate of the handler with the approval of the guide dog school, and one dog was withdrawn by the guide dog school for reasons unknown. The remaining three dogs that were returned, although successfully matched, were ultimately returned by handlers who had made informed choices to accept these dogs when the guide dog school discussed potential problems at the time of matching, and these problems were the reason for return. Two of these dogs were returned for dog distraction/aggression and one for an ongoing health problem.

Overall, handlers claimed to be very emotionally attached to 18 of the 20 dogs in this group. Regarding the other two dogs, one handler was moderately attached (temporary match), and the other, who returned her dog because of its poor health, felt guilty for not bonding more with a dog she had not realized at the time was too sick to work effectively.

##### Combination 4: Mismatched and Returned

Twenty-three dogs (19.5%) were returned for being considered to be poorly matched by the handler. Reasons provided for this included the dog having poor social/home behaviors, limited capacity to work (work rate), problems with specific-guiding tasks (including speed), and having poor coping skills. However, as shown above, more dogs were thought to be mismatched (26.3%, *n* = 31) than were actually returned for this reason. None of the three handlers who returned their dogs because of, or related to, their own behavior considered these dogs to be mismatched.

#### Relationships between the Combinations

**Figure 3:**
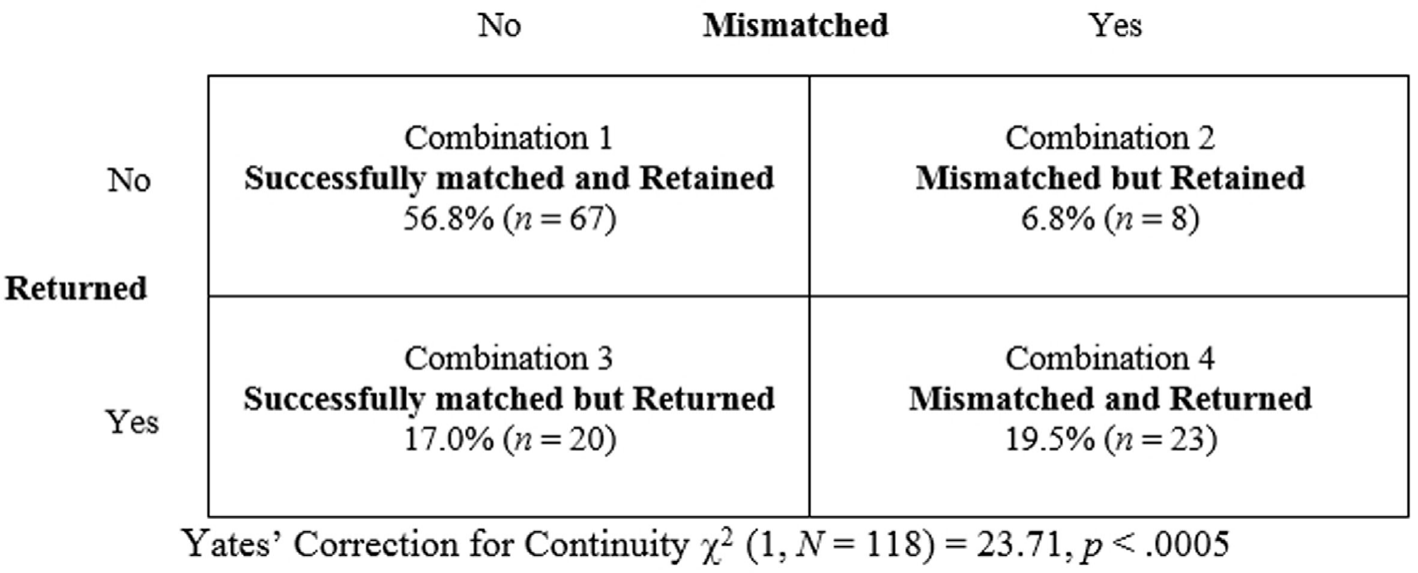
**Association between matching success and dogs that are returned or retained**.

Frequencies for the four combinations to which the handlers assigned their dogs are illustrated in Figure 3. A chi-square test for independence was conducted to examine these relationships. A significant result was obtained using Yates’ Correction for Continuity [χ^2^ (1, *N* = 118) = 23.71, *p* < 0.0005] suggesting that the proportion of dogs that was returned for being mismatched (74.2% of all mismatched dogs) was significantly different from the proportion of dogs that was returned although successfully matched (23% of all successfully matched dogs). A calculation of the odds ratio indicated that dogs were 9.6 times more likely to be returned if deemed mismatched.

#### Duration of the Partnership

Seventy-nine (66.9%) of the 118 dogs in the sample had reached the end of the working partnership (retired or returned) with a particular handler (Table 1). The duration of the partnership is calculated only on these 79 dogs, as the sample as a whole does not reflect the full working life of all partnerships. The 79 dogs previously employed as a guide worked from as little as 1 month to as long as 156 months^7^ (13 years) with an average working life of 4.7 years (M = 55.9 months, SD = 46.0). Handler defined successful partnerships (*n* = 53), lasted from one month to 13 years, with an average service duration of 6 years (M = 72.3 months, SD = 42.8), and the largest number of dogs (mode) were retired at about 10 years of age (norm). Unsuccessful partnerships (*n* = 26) (inclusive of three dogs that were not returned) lasted from 1 month to 11.5 years (138 months), but dogs were returned on average at less than 2 years (M = 22.2 months, SD = 32.2), with the largest number (mode) being returned after 3 months. Dogs that were returned for being unsuccessfully matched (*n* = 23) worked from 1 month to 6 years (72 months) and were returned on average at just over 1 year (M = 12.6 months, SD = 14.7). All dogs that were returned for being unsuccessfully matched were returned within 2 years, with the exception of one dog that worked for 6 years. Excluding this dog, dogs that were returned for being unsuccessfully matched worked on average for just under 10 months, and the largest number were returned after 3 months.

#### Trends between Dogs

Concerning the relationships handlers had with their dogs, a recurring trend of “first dog best—second dog worst,” with little apparent difference between the first and third dogs, was found. As this pattern may be of interest to the guide dog industry, this “second dog syndrome” was further investigated. The focus is on the trends between the first three dogs only, as these dogs comprise the majority of dogs (82.2%) in the sample.

**Figure 4:**
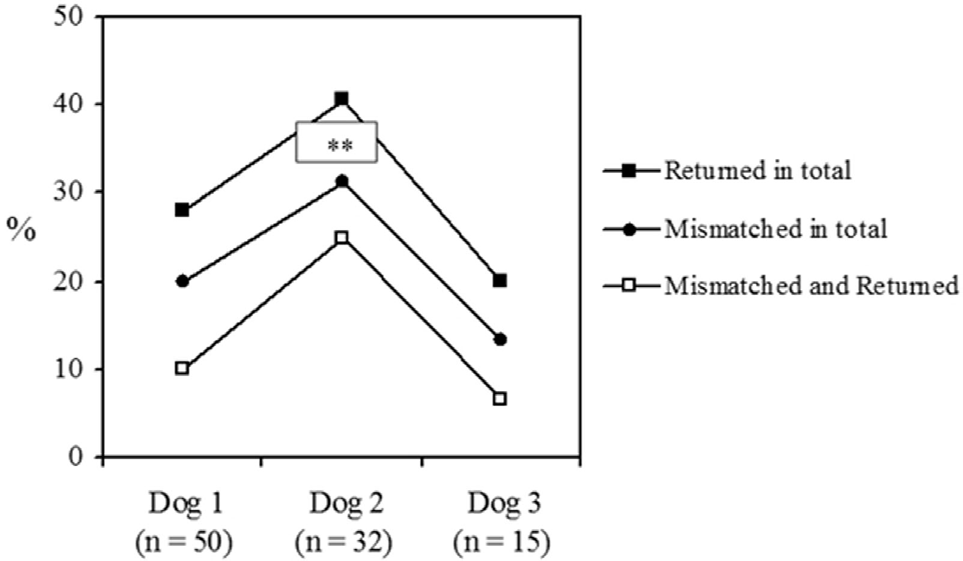
**Comparisons of the relationships between the first, second, and third dogs concerning the percent of dogs returned, mismatched, and those returned for being mismatched**. **Denotes the significant relationship between the second dogs that are returned for being mismatched and those that are retained (*p* < 0.004).

When accounting for the proportion of dogs at the time the study was conducted, the number that were (a) returned, (b) mismatched, and (c) mismatched and returned were all highest for second dogs and lowest for third (Figure 4). Odds ratios indicated that the likelihood of a dog being (a) returned and (b) mismatched was nearly twice (1.8) as high for second dogs as first dogs, and second dogs were three times more likely to be (c) mismatched and returned than first dogs. Third dogs were around three times less likely than second dogs to be (a) returned (2.7) or (b) mismatched (3.0), and nearly five times less likely to be (c) returned for being mismatched. Only half of the mismatched first and third dogs were returned, but mismatched second dogs were returned four times as often. None of these trends reached statistical significance on chi-square analyses. However, second dogs were significantly (four times) more likely to be returned if mismatched than retained (Fisher’s Exact Probability test *p* < 0.004).

In light of this, it is not unexpected that the working life of dogs that had been retired or withdrawn before the study commenced (*n* = 79) is shortest in second dogs (Table 1). Independent samples *t*-tests revealed that second dogs (M = 44.1, SD = 47.3) were used for significantly less time than first dogs [M = 69.7, SD = 41.2, *t*(53) = 2.09, *p* = 0.042, η^2^ = 0.08]. No significant difference was seen between second and third dogs (M = 65.8, SD = 46.5, η^2^ = 0.05). However, this was likely due to the small number of third dogs that were not in current use in the sample (*n* = 8).

#### Defining Matching Success

Based on the study findings, defining matching success is not clear-cut. The results indicate that a substantial number of handlers return dogs (17.0%, *n* = 20) for reasons that do not pertain to being mismatched, mainly for the dogs’ poor health. The results also suggest that more dogs were considered mismatched (26.3%, *n* = 31) than were returned for problems arising from these mismatches (19.5%, *n* = 23). As a goal of this research was to identify what factors were important in creating a successful match, it seemed sensible to focus on whether a dog was considered mismatched *per se* as opposed to whether it was returned for being mismatched.

**Figure 5:**
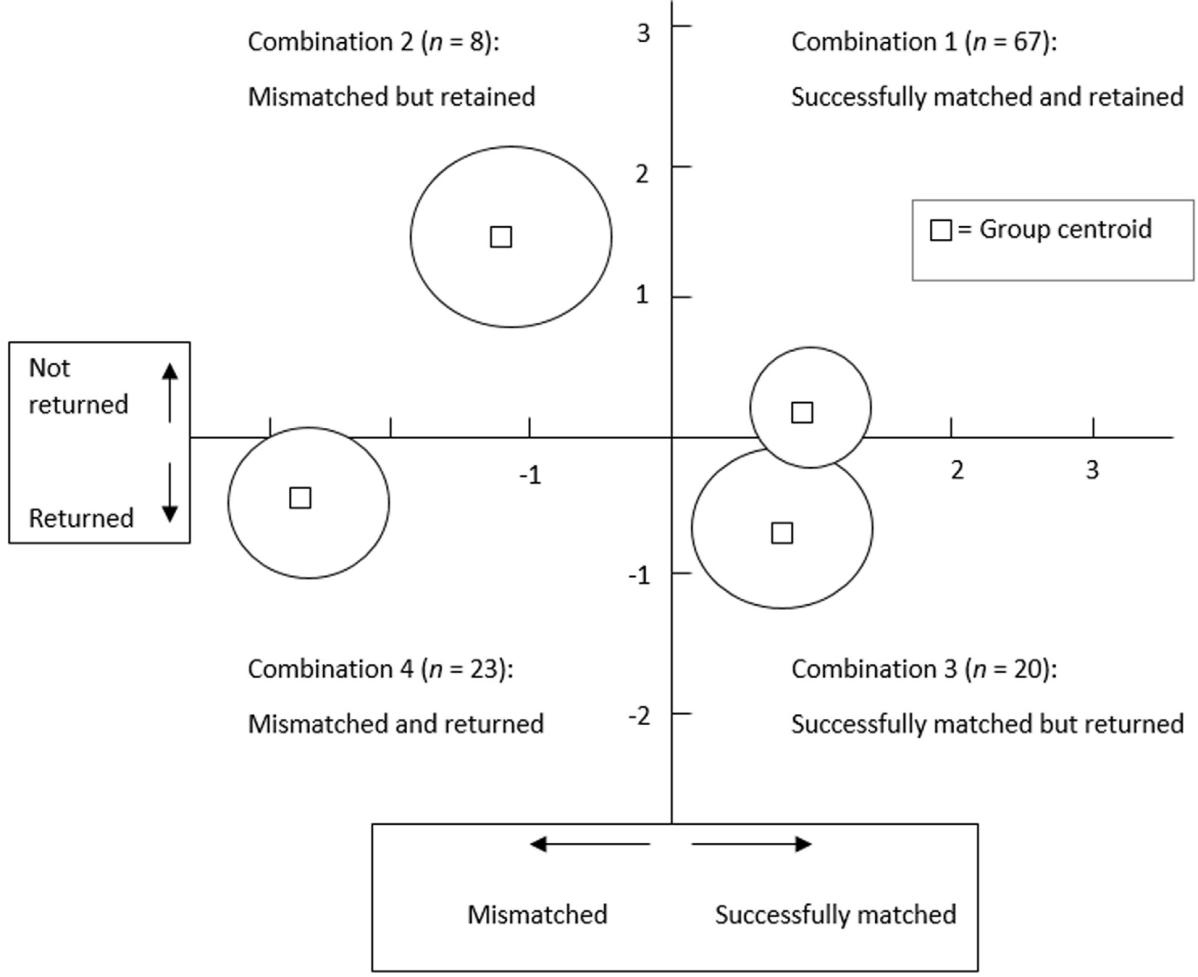
**A discriminant function analysis plot showing estimation of the group centroids and the corresponding confidence circles for matching success for Combinations 1–4**. Note: the confidence circles were calculated as per Maxwell (29).

A discriminant function analysis was conducted using the data relating to the four matching success/outcome categories to check that the above classification decision was viable. Although a significant Box’s *M* value indicted that assumptions of equality of covariance matrices were not met, the results (Figure 5) suggest that there were three significantly distinct groups (χ^2^ = 170.57, *df* = 36, *p* < 0.0005): Combination 2, Combination 4, and Combinations 1 and 3 combined. In effect, the dogs deemed to be mismatched (Combinations 2 and 4) were considerably different from those that were not (Combinations 1 and 3). There were no meaningful differences between dogs that were considered successfully matched that were retained (Combination 1) and those that were returned (Combination 3). Dogs that were considered mismatched but retained (Combination 2) appeared to differ somewhat from those that were considered mismatched and returned (Combination 4). However, the number of dogs in Combination 2 (*n* = 8) was very small, and the decision to combine these dogs with the other mismatched dogs in Combination 4 was justified based on the qualitative data described in “Classification of Outcome.” Hence, it was decided that a reasonable definition of matching success was whether dogs were considered to be mismatched or not by their handlers.

### Rematched Dogs

Just over a third (*n* = 17) of the participants had used or were using dogs that had been matched with at least one other previous person. This scenario is common as dogs are returned to guide dog schools for a variety of reasons that do not preclude them from being rematched to others. These reasons include ill-health (of the person), emigration, or changes in individual mobility needs and/or family dynamics.

The majority (84%) of participants said that they were or would be content to be matched with a dog that had been previously used as a guide by another person, although caveats included “it is OK, as long as you know the dog’s history” and “people need to know that it may take longer to bond [with the dog].” Some participants preferred dogs that had been used previously as a guide, as these dogs tended to be more mature and, therefore, less rambunctious than dogs fresh out of training, or “had been round the block and knew a thing or two about guiding.” Eight participants (16%) stated they would not be happy if offered a dog that had been previously used as a guide because they were concerned that it may be more likely to have behavioral problems or take longer to adjust to a new home/working environment.

Twenty (17.2%) of the 118 dogs in the sample had been returned to the guide dog school by their previous handlers, and ultimately, 15 of these 20 rematches were successful. However, according to the participants in this study, four of these 15 dogs did not have a successful partnership with the first person they were rematched to, but did on a subsequent match, and three of these 15 dogs had the opposite experience. Of the five dogs that were not rematched successfully, three were eventually withdrawn; one for extreme excitability, one for marked aggression to other dogs, and one for a musculoskeletal problem. The other two dogs that were not rematched successfully, although currently working, were being considered for return, as one exhibited the same problem that its previous handler returned it for (scavenging) and the other developed an unrelated health issue (skin problem).

## Discussion

The purpose of this study was to investigate the relationship between handlers and their guide dogs to understand, from the handlers’ perspective, why some partnerships worked while others did not, to improve the outcome of the matching process. Defining matching success is not clear-cut. Not all dogs that were returned were considered by their handlers to have been mismatched, and not all dogs retained until retirement were thought to have been good matches. The latter finding suggests that some handlers were retaining what they considered to be a poorly matched dog, which could have detrimental effects on both the handlers’ and the dogs’ quality of life.

This study measured the success of the match based on whether handlers thought the dog was mismatched or not. Ratings of good and bad behaviors and physical characteristics described what qualities handlers found attractive and unattractive in guide dogs in general, and how they related to work (mobility-related) versus NW (home/social) situations, and were primary used to describe the data in a meaningful way. While there are theoretical grounds to believe that a handler is more similar to themselves than another handler in how they perceive a dog, each human–animal relationship is unique and the decision to treat each handler-dog pairing as independent observations was supported by statistics (i.e., insignificant ICC value and average number of dogs used close to only two). However, although not detectable in the present study, it is possible that some handlers might be more likely to return dogs than others. For example, a person who may have had a specific problem with one dog might pay more attention to the same issue in a subsequent dog, and this should be taken into account during the matching process.

Although several studies have described peoples’ attitudes toward guide dogs (1, 23, 30, 31), there appears to be little data available on the reasons why some matches fail. This may be because some guide dog schools compete with others for clients. However, it would benefit those involved with guide dogs if this information was more freely available.

### The Outcome of the Partnership

Most dogs in this study were successfully matched. Partnerships ended for one of the three reasons: (1) the dog retired, (2) it was returned (whether mismatched or not), or (3) it died. Over a third of dogs were returned in total, primarily for problems concerning the dogs’ behavior, followed by canine health problems. In addition, three handlers returned their dogs for personal or social reasons. More dogs were returned for behavior problems relating to work than for NW. However, the largest single behavior problem that dogs were returned for was poor home/social behavior. It would be advantageous for guide dog schools to pay equal attention to working and non-working behaviors when training dogs and making matches, as in addition to this finding, Lloyd et al. (24) and Lloyd (32) found that factors relating to both the working and the non-working relationship appeared to be significant predictors of matching success.

Just over a quarter of dogs were considered to be mismatched, but only a one-fifth of dogs were returned for this reason. Reasons for dogs being returned or considered to be mismatched related more to the dog than the handler, and problems were behavioral more than physical. More dogs were considered mismatched for reasons that pertained to work than for NW. This discrepancy was due to a number of dogs being returned, despite being considered successfully matched, for health reasons. The probability of a mismatched dog being returned was several fold higher than for successfully matched dogs, and as may be expected, the reasons that dogs are returned (whether mismatched or not) correspond with what handlers said they liked the least about their dogs. It should be appreciated that matching is not absolute; some unsuccessful matches had good points, and few successfully matched handlers claimed to have a perfect dog. However, some handlers kept dogs that they thought were mismatched because they were emotionally attached to the dog, had enough vision to compensate or were inexperienced. This was more likely to happen for first-time dogs than second ones, which is discussed below.

Successful partnerships lasted for an average of 6 years, with the largest number (mode) of dogs being returned after 10 years of service. This is lower than the average of 7 years reported by Nicholson et al. (22). However, the present study included dogs that had previously worked with other handlers and had been rematched, and it is possible that Nicholson et al. (22) were referring to successfully matched dogs that had worked with only one handler. A recent paper by Caron-Lormier et al. (33) who investigated aging in guide dogs also recorded the length of the dogs’ working life. The researchers found that 84% of working guide dogs were able to function as guide dogs until they had worked for 8.5 years, when they retired. However, this sample excluded dogs that were withdrawn for behavioral reasons and so methodological issues make it difficult to compare this study with the present study.

In the present study, excluding one dog that worked for 6 years before being returned for behavioral problems, mismatched dogs worked for 10 months on average, but the largest number (mode) were returned after just 3 months—presumably before an emotional bond had fully developed. Therefore, a handler who is frustrated with a new partnership should be informed that the working and the non-working relationship might take longer than this to improve, possibly from 6 months up to a year (34). These findings are similar to those of Fuller (35) who reported that most unsuccessfully matched dogs were returned within the first year, and that several dogs were returned for behavioral problems after 5 years of use. Although not stated by Fuller (35), it is possible that these late returns were due to replacement dogs becoming more readily available at that time. Fuller (35) indicated that returns were due to handler-related reasons one-third of the time, and the remainder for dog-related reasons, with physical incapacity or death being the major factor in both categories. Both Fuller (35) and the present study suggests that the number of returns because the handler has personal or social problems is small, but results differ in that Fuller (35) reported more dogs stopped working for health (59.4%) than for behavioral problems (7.4%). Adjusting for the inclusion of the human’s physical incapacity or demise as reasons for partnerships to end, the number of dogs returned for behavioral problems in the present study (22.0%) was double that of Fuller (35) figures (10.2%). This finding supports Ireson (15) theory of dogs being returned less often by previous generations of guide dog handlers. However, it could not be ascertained what the specific behavioral problems were in the Fuller (35) study nor whether they related to work and/or NW.

The number of dogs in the present study that were unsuccessfully matched (26.3%), and returned for this reason (19.5%) is comparable to the 25.4% that were withdrawn in Nicholson et al. (22) UK study. These researchers did not define the reasons dogs were withdrawn. However, conversations with guide dog professionals at symposiums attended during the course of this research, suggest that the current findings reflect the outcome of guide dog programs around the world in terms of numbers, reasons, and trends. These numbers also relate to the 16.0% of pet dogs adopted from animal shelters in New Zealand returned for unacceptable behaviors (36). Breakdowns in the owner–pet relationship may occur because the owner has unrealistic expectations of the role of a pet and/or is ignorant of breed-specific behaviors, and the time and money required for maintenance (37). Although guide dogs are provided free of charge in many countries, and guide dog schools protect the dogs’ welfare in cases of hardship, the use of guide dogs can be expensive. Intriguingly, the return rate of dogs is analogous to the 29.3% (38) abandonment rate of assistance technology devices (excluding dogs). Scherer (38) found that the most influential factor was a change in needs/priorities of the user, but that the user’s input into the selection of the device was important for a good outcome.

Only the main reasons dogs were returned were identified in the present study. However, it is likely that more than one problem contributed to their return. Future research could establish if handlers return dogs due to an accumulation of problems and if these problems interrelate. For example, if a dog’s level of anxiety increases during its working life, the pace at which it walks may increase resulting in an incompatible match. In addition, an anxious dog may be overly sensitive and have poor social/home behavior, that is, multiple problems stemming from the same underlying concern. Physical attributes such as breed or size may also be linked to behavioral problems. For example, large dogs may be too strong if inclined to pull through the harness. A breed-specific behavioral problem, which confirms the importance of educating handlers about their choices and expectations, is epitomized by the handler who thought that everything would be alright if only her next dog was a Standard Poodle. However, when this eventuated, the Poodle was described as “dizzy” and “unfocussed,” and was subsequently returned for this and other behaviors common within the breed.

Only one dog in the present study was withdrawn primarily for a skin problem, and one was being considered for return as the problem was severe, but “skin” was frequently mention as problematic. Furthermore, fieldwork associated with this research suggested that more dogs appeared to have health issues in recent times than in the past, especially skin problems, This is important, as Caron-Lormier et al. (33) found that of 14 groups of health problems in 8 of the most common breed (plus “other”) of guide dogs, skin problems had the greatest impact on reducing working life, by an average of 5 years. There was no discernible rise in the small number of dogs being returned for health problems over the years dogs were used in the present study. However, only the main reasons for return were considered and it is possible that health issues were also a major, albeit secondary, concern. Musculoskeletal disorders were the most common reason for dogs being withdrawn prior to retirement in both the Caron-Lormier et al. (33) and the present study. In-depth examinations of guide dog schools’ records to identify health problems and establish if these are a growing concern would be invaluable for making matching decisions, and for the breeding program if these conditions were heritable.

Not being able to walk at their preferred walking speed was also of concern to handlers in the present study as being forced to walk too fast can be frightening, and too slow frustrating. However, few dogs were returned primarily because of a speed mismatch. This suggests that instructors are adept at matching for speed because (a) they are aware of its importance and (b) because speed is more quantifiable (for human and canine) than many other matching criteria.

Of interest, is that many handlers appeared to feel the need to defend their preferences for physical traits in their dogs, including why they wanted a dog to be good-looking, and how the dog felt to touch (tactility). This concept is supported in a focus group discussion on guide dog usage (34) concerning the general public sometimes being insensitive to people with a visual disability preferring a dog of a certain color.

Another interesting finding was that three of the handlers who returned their problem dogs did not feel that they had been mismatched because the instructors discussed the potential for these particular problems at the time of matching, thus empowering the handlers to make informed choices. A similar concept exists when handlers do not consider dogs with health problems to be mismatched, if these problems were unforeseen. Conversely, handlers were upset and angry if they subsequently discovered they had been matched with a dog that had been returned by a previous handler for a problem that the new handler was unaware of. Candidness is arguably the best policy for client satisfaction and the opportunity for person and dog to work through problems together may actually strengthen the bond. An important aspect of the relationship is cooperation between dog and handler (39) with the handler being in control for some tasks but permitting the dog to also use its initiative in making suitable guiding decisions. Allowing the dog some freedom of choice might help a guide dog reach its potential in its working role; it is feasible that dogs that are not afforded some measure of choice have less chance to develop self-control (16).

### Rematched Dogs

Problems between people and their guide dogs are common, and, as with pet ownership, relationships often break down. However, a problem for one person may not be a problem for another, and dogs are returned for reasons that do not preclude them from working with a subsequent handler, for example, ill-health of the previous handler. Twenty dogs in this sample had been rematched, some twice, with a success rate of 75% (i.e., comparable to that of all the dogs in the sample). The remainder were withdrawn from the program and rehomed as pets.

No patterns emerged regarding what might constitute success, as both the successfully and the unsuccessfully rematched groups had been rematched for a variety of issues. However, it is apparent that the rematching of dogs is an appropriate use of resources. Most participants were happy with the notion of being matched with a previously employed dog, provided they were aware of the dog’s history. Fuller (35) commented that some returned dogs did well when rematched, although he provided no other details. Ledger and Baxter (40) concluded that successive owners of the same pet dog, which was repeatedly adopted from a UK animal shelter, reported different behavioral problems due to different attitudes and perceptions. Pet owner attitudes are believed to directly affect behavioral problems in dogs, particularly (what was previously known as), dominance aggression and displacement/excitement behaviors (41, 42). For these reasons, it would be interesting to compare the experiences of successive handlers of rematched dogs.

### Second Dog Syndrome

An unexpected finding was that handlers described inferior relationships with their second dogs compared to their first and third dogs. First-time dogs were favored the most, but there was little apparent difference between the first and third dogs. The term “second dog syndrome” was coined for this discussion (32).

It is feasible that fewer first dogs were rejected for the same reasons that handlers did not return mismatched dogs, as discussed above. These include expectations being lower due to people not knowing what to expect (having had no other dog to compare), having enough vision to compensate for dogs that did not excel as mobility aids and being more emotionally attached to these dogs. As the use of more than one guide dog is common, it would be interesting for future research to compare people who had only used one dog with those who had used multiple dogs to ascertain if experience has an effect on matching success.

It is understandable why there should be a “first-dog” effect in the handlers’ affections, as this dog was the one to initialize and/or improve independent mobility, thus being the catalyst for life changing events. Another possible explanation provided by Lloyd et al. (21) is “distortion of memory” where handlers may have forgotten the boisterous behavior of the first dog when it was young, and are comparing a youthful, exuberant second dog to that of the first dog at the end of the partnership when it had matured. It is also possible that guide dog schools might match second-time dog users with less than optimal dogs in the belief that the handler will cope with a dog that is more of a “challenge.” However, anecdotal evidence from this study suggests that some dogs that were returned were successfully matched to first time as well as experienced users, and third dogs were less likely to be returned or deemed to be mismatched than second dogs. Regardless, knowing that a second dog is likely to be perceived as second best is useful knowledge for guide dog instructors to help prepare clients who are about to receive a second dog. Following on from Lloyd et al. (21) work, Ward and Peirce (43) created a client- driven information resource for second time guide dog applicants to aid in the transition of dogs.

## Conclusion

This research serves to increase awareness of what is happening in guide dog partnerships post-qualification. This research is intrinsically important for the guide dog industry in several ways. It examined, in a real-life setting, the outcome of the matching process; a process that is widely practised, but little assessed, and highlights the need to consider not only working behaviors but also social/home behaviors when making matching decisions. Understanding what makes a successful partnership is becoming increasingly important as there has been a steady increase in the number of handler-guide dog teams graduating around the world, as well as in the number of other service (or assistance) animals. Guide dogs are expensive to produce, as well as being expensive in personal terms for all concerned if a match is unsuccessful, and it may be assumed that the number of unsuccessful matches is likely to increase relative to the total number of matches made. Although feelings at the end of the partnership were not a focus of this study, nearly two-thirds of participants had used more than one dog. As the transition from one dog to the next is a recurring feature, handlers probably experience the end of more relationships than the average pet dog owner (22). Retiring a guide dog is not only difficult for the handler but also for the handler’s family and friends (21) and no doubt also for the dog. Every participant in this study was matched successfully at least once, and most dogs that were rematched went on to have a successful relationship with a different handler. This shows that the success of the handler and guide dog partnership does not solely depend on either the person or the dog, but relies on the interplay between them, that is, the match.

## Ethics Statement

Massey University Human Ethics Committee. The study was carried out in accordance with the recommendations of Massey University Human Ethics Committee with written informed consent from all subjects. The participants in the study were vision impaired.

## Author Contributions

All the authors (JL, CB, SL, and KS) on this publication have contributed to the conception and design of this work. The first author (JL) undertook the research and wrote the article with the approval of the other authors (CB, SL, and KS) who have critically revised the content. All the authors (JL, CB, SL, and KS) agreed to be accountable for the content.

## Conflict of Interest Statement

The authors declare that the research was conducted in the absence of any commercial or financial relationships that could be construed as a potential conflict of interest.

## Footnotes

^1^A sexually intact working guide dog is rare; permission was granted from the participant who used this dog to be identified in this manner.

^2^A discriminant functional analysis (Defining Matching Success) supported that “matching success,” as defined by the handler, was a suitable outcome (dependent) variable for the ICC analysis.

^3^Dogs that stopped working at age eight and older were classified as “retired,” including dogs that died after this age.

^4^The term “returned” pertains to a dog that is less than 8 years old, which did not succeed as a guide for a particular handler. Many dogs that are returned are re-matched to other people with varying degrees of success. Dogs that were returned but not re-matched are classified as “withdrawn.”

^5^“Emotional” is defined as a psychological response that could not be classified as either behavioural or physical.

^6^The category “handler” incorporates the effects of Dahlgren and Whiteheads’ (28) socioeconomic model where the handler’s decision regarding the return of a dog may have been influenced by social or environmental factors not of the handler’s choosing, such as a dog not being wanted by family or work-mates.

^7^Only one dog worked for 13 years, and this was an unusual case that had a very limited workload for the last few years of its working life.
