# Supplementary material for: An Investigation of the Complexities of Successful and Unsuccessful Guide Dog Matching and Partnerships
Source: Front Vet Sci. 2016 Dec 16;3:114. doi: 10.3389/fvets.2016.00114 (PMC5159482; doi:10.3389/fvets.2016.00114)
Supplement: Supplementary file 1 [file Data_Sheet_1.doc]

# Guide Dog & Handler Survey

Introduction to Interview

- Introduce self as researcher.
- Ask if this is an appropriate time to conduct the telephone call. If not, arrange for a suitable time and record below. If suitable, read the following…

*Thank you for agreeing to participate in this survey designed to help understand the changes that using guide dogs makes in peoples’ lives and what constitutes a good match. The questionnaire consists of six sections that ask about: you, your guide dog/s and pets, your expectations and outcomes of using a guide dog, mobility, compatibility with your dog/s, the dogs’ affects on overall quality of life, and will finish up by asking for your suggestions concerning the services you receive from GDS.*

*I anticipate that the entire questionnaire will take between one and two hours to complete, depending on how many guide dogs you have used. I appreciate that this is a very long time, so please feel free to let me know when you have had enough and we can reschedule the remainder at a time convenient to you.*

*Please remember that I am not an employee of the RNZFB, and everything you tell me will be combined with information from other guide dog handlers so that you cannot be identified, unless you give me permission to do so. Therefore, you may be as open as you wish.*

*Are there any questions you would like to ask before we begin?*

New time for telephone/face to face interview (circle): ________________________________________

Note: For ease of use, the questionnaire has been written using the present tense. However, the past tense should be used throughout the questionnaire when asking about a previous dog/s or experience, and the present tense for previous or current dog or experience, as appropriate.

**Section 1 - General**

This first section asks general questions about **you, your vision, guide dogs** and **pets**.

What is your **gender**? male ⬜ female ⬜

What **age** are you? ______________________________________________________________________

What **ethnic group** do you identify with? ____________________________________________________

What is the underlying cause of your **sight impairment or blindness**? _____________________________

How **old** were you when you **lost your vision**? ________________________________________________

If vision loss was **acquired** (adventitious), was this loss gradual ⬜ or sudden ⬜ n/a ⬜

What year did you become a **member** of the RNZFB? __________________________________________

Are you **currently** using a guide dog? yes ⬜ no ⬜

Are you currently on the **official waiting list** (OWL) for a new dog? yes ⬜ no ⬜

Please explain why you are on the OWL: ______________________________________________ n/a ⬜

Please explain why you are not on the OWL: ___________________________________________ n/a ⬜

Have you **previously** had a guide dog? yes ⬜ no ⬜

If yes, what is the **total number of dogs** used, including your current dog? ________________________

Were you aware that you were **entitled to apply** for a guide dog? yes ⬜ no ⬜ (why not?) ____________

What/whom gave you the **idea to apply** for a guide dog? ______________________________________

Did you **apply** for a guide dog **to please**: yourself ⬜ others ⬜ both ⬜________________(mainly whom)

## *‘Ideal Dog’ – Standards of Guide Dog Use*

The next set of questions asks about your **expectations** regarding guide dog use.

Knowing what you know now, please imagine your *ideal* (hypothetical) guide dog:

What would you expect your ‘ideal dog’ to do for you in terms of **travel** (mobility)?

Please answer in order of importance: ______________________________________________________

What would you expect your ‘ideal dog’ to do for you in terms of **social-interactions/function**?

Please answer in order of importance: ______________________________________________________

What would you expect your ‘ideal dog’ to do for you in terms of **companionship?**

Please answer in order of importance: ______________________________________________________

Do you have any **other** expectations of guide dog use? If so, what are they?

Please answer in order of importance: ______________________________________________________

Of the following categories: travel, social-interactions/function, companionship and other, please **rank** in order of most to least important: ________________________________________________________________

Knowing what you know now:

What **behavioural** characteristics would your ‘ideal’ guide dog have?

Please answer in order of importance: ______________________________________________________

What **physical** characteristics (including health), would your ‘ideal’ guide dog have?

Please answer in order of importance: ______________________________________________________

Of the following categories: behaviour and physical please **rank** in order of most to least important: ______________________________________________________________________________________

***Previous Pets***

The last items in this section refer to any **previous pets** you may have had.

Before you got a guide dog, were you ever **responsible** for a pet or pets? yes ⬜ no ⬜

Before you got a guide dog, did you have an **affinity** with animals? yes ⬜ no ⬜

What **age** were you when you got your first pet? _________________________________________ n/a ⬜

If applicable, what was the pet **most important** to you **during childhood** (< 12 years)? __________ n/a ⬜

*End of Section 1*

**Section 2 - Information on Each Guide Dog Used**

The questions you have just completed end the first section. The next set of questions asks a variety of questions about your **first guide dog**, and, where applicable, these questions will be repeated for **subsequent dogs** in the order you used them.

Is this dog your **current** guide dog? yes ⬜ no ⬜

Are you the **legal owner** of this dog, or does the dog belong to GDS?

Did you and this dog **graduate** as a working team? yes ⬜ no ⬜

Which **country** was this dog received in? ___________________________________________________

Dog **order** (ascending): ____________________________________________________e.g. 1^st^ of 3 dogs

Dog’s **name**: _________________________________________________________________________

Dog’s **breed**: _________________________________________________________________________

Dog’s coat **colour**: _____________________________________________________________________

Dog’s **sex** (neutered): ___________________________________________________________________

What year was this dog **born**? ____________________________________________________________

What year did you **receive** this dog? _______________________________________________________

How long has this dog **worked** with you? ___________________________________________________

What was the main reason this **partnership ended**? ______________________________________ n/a ⬜

# How long were you on the **waiting list** for this dog (provisional and official)? _______________________

Was this wait: too long ⬜ too short ⬜ about right ⬜

# ***Training & Workload***

Did you have a choice of **venue** where you trained with the dog i.e. home or elsewhere? yes ⬜ no ⬜

Where were you trained? _________________________________________________________________

On a scale of 1-10, overall how **satisfied** were you with the amount and quality of **training** you received?

not at all 1 2 3 4 5 6 7 8 9 10 completely satisfied

Comment? ____________________________________________________________________________

## Which of the following environments do you require this dog to work in?

rural yes ⬜ no ⬜

semi-rural yes ⬜ no ⬜

residential yes ⬜ no ⬜

semi-business/quiet city yes ⬜ no ⬜

busy city yes ⬜ no ⬜

other (specify): yes ⬜ no ⬜

What is the main environment you required this dog to work in? ________________________________

What **quantity of work** do you require this dog to do? low ⬜ medium ⬜ high ⬜

Does the dog cope well with this **workload** (environment & quantity)? yes ⬜ no (specify) ⬜__________

***Dog’s Behavioural Characteristics***

What is *good* regarding this dog’s behaviour, in order of importance? _____________________________

What is *bad* regarding this dog’s behaviour, in order of importance? ______________________________

What has been done to resolve the problem/s? **__________________________________________** n/a ⬜

Is the problem/s resolved? yes ⬜ no ⬜ somewhat ⬜ n/a ⬜

***Dog’s Physical Characteristics***

What is *good,* physically, about this dog, in order of importance? ________________________________

What is *bad,* physically, about this dog, in order of importance? _________________________________

What has been done to resolve the problem/s? **__________________________________________** n/a ⬜

Is the problem/s resolved? yes ⬜ no ⬜ somewhat ⬜ n/a ⬜

***Outcomes of Expectations***

Earlier, I asked about your **expectations** regarding guide dog use; at the time you used this dog, were your expectations regarding…

Travel (mobility): not met ⬜ somewhat met ⬜ met ⬜ exceeded ⬜ n/a ⬜

Social-function: not met ⬜ somewhat met ⬜ met ⬜ exceeded ⬜ n/a ⬜

Companionship: not met ⬜ somewhat met ⬜ met ⬜ exceeded ⬜ n/a ⬜

Other: not met ⬜ somewhat met ⬜ met ⬜ exceeded ⬜ n/a ⬜

Behaviour: not met ⬜ somewhat met ⬜ met ⬜ exceeded ⬜ n/a ⬜ Physical/health: not met ⬜ somewhat met ⬜ met ⬜ exceeded ⬜ n/a ⬜

Were there any **benefits** you received from using this dog that you had **not expected**? yes ⬜ no ⬜

If yes, what were they? _____________________________________________________________ n/a ⬜

How did/do your **friends and family** feel about this dog? _______________________________________

***Compatibility***

The following questions are designed to measure how **compatible** overall you and this dog are:

## On a scale of 1-10, how much have you bonded with this dog i.e. an emotional attachment *between* you and the dog?

not at all 1 2 3 4 5 6 7 8 9 10 extremely well bonded

If applicable, how long did it take you to bond with this dog?________________________________ n/a ⬜

## On a scale of 1-10, how would you rate your working relationship with this dog i.e. the dog’s work and how this affects your mobility?

very poor 1 2 3 4 5 6 7 8 9 10 very good working relationship

If applicable, how long did it take to form a good, working relationship? ______________________ n/a ⬜

On a scale of 1-10, how great was your **need** to get this dog?

very low 1 2 3 4 5 6 7 8 9 10 extremely high need

On a scale of 1-10, how **motivated** were you **to acquire** this dog?

not at all 1 2 3 4 5 6 7 8 9 10 extremely motivated to acquire

On a scale of 1-10, how **motivated** are you **to** **succeed** with this match?

not at all 1 2 3 4 5 6 7 8 9 10 extremely motivated to succeed

On a scale of 1-10, how well can you **control** this dog (working and social behaviour)?

not at all 1 2 3 4 5 6 7 8 9 10 extremely well (controlled)

## On a scale of 1-10, how emotionally attached are you to this dog i.e. one-way relationship unlike the bond which implies a two-way relationship?

not at all 1 2 3 4 5 6 7 8 9 10 extremely attached

##

On a scale of 1-10, how **well suited** are you and this dog?

not at all 1 2 3 4 5 6 7 8 9 10 totally suitable

On a scale of 1-10, how satisfied are you with this dog’s affects on your **social-interactions/function**

not at all 1 2 3 4 5 6 7 8 9 10 completely satisfied

On a scale of 1-10, how satisfied are you with this dog’s qualities as a **companion?**

not at all 1 2 3 4 5 6 7 8 9 10 completely satisfied

On a scale of 1 – 10, overall, how **satisfied** are you with this dog?

not at all 1 2 3 4 5 6 7 8 9 10 completely satisfied

On a scale of 1 – 10, overall, how successful do you feel this **match** is?

not at all successful 1 2 3 4 5 6 7 8 9 10 totally successful

Would you consider this dog to have been mismatched? yes ⬜ no ⬜

Was this dog returned to GDS? yes ⬜ no ⬜

Why did you **not return** this dog, which you considered to be **mismatched**? ___________________ n/a ⬜

### Or:

Why did you **return** this dog, which you did **not** consider to be **mismatched**? _________________ n/a ⬜

### In your opinion, why is this **match working**? ___________________________________________ n/a ⬜

### Or:

### In your opinion, why is this **match not** **working**? ________________________________________ n/a ⬜

# ***At the End of the Partnership***

The next set of questions pertains to issues that arise at the **end of the partnership**. If you are currently working with your first dog, please feel free to project how you think you may feel at this eventuality.

Select option: actual feelings for previous dog ⬜ projected feelings for current dog ⬜

What will happen to this dog after the partnership ends?________________________________________

Will you keep in touch with the dog if possible? yes ⬜ no ⬜

How will you feel at the end of this partnership? ______________________________________________

How long will the loss of this dog put you off applying for a replacement? _________________________

Comment: ____________________________________________________________________________

And the final question regarding the end of the partnership:

How will the end of this partnership affect your relationship with your next dog? _______________ n/a ⬜

***Demographic & Visual Status***

I’d like to now ask you some more **demographic questions** concerning the time you **obtained** this dog, including questions about your home and work situation, and vision. Firstly:

On a scale of 1-10, what was your **relationship** with your guide dog instructor?

very poor 1 2 3 4 5 6 7 8 9 10 very good relationship

Did you **move house and location** during the two years before or after obtaining this dog? yes ⬜ no ⬜

What **age** were you were you obtained this dog? ______________________________________________

What was your **marital status**? ____________________________________________________________

What was the highest level **education** you received? ___________________________________________

What was your **occupation**? ______________________________________________________________

If **employed**, were you: full-time ⬜ part-time ⬜ n/a ⬜

What kind of **housing** did you live in? ______________________________________________________

Was the **property**: rented ⬜ bought ⬜ live with parents ⬜ other (specify) ⬜_____________________

How many **adults** were in the household? ___________________________________________________

How many **children** were in the household? _________________________________________________

Excluding this guide dog, did you own any **pets**? yes ⬜ no ⬜

If yes, how many **dogs**?_____________________________________________________________ n/a ⬜

If yes, how many **cats**?_____________________________________________________________ n/a ⬜

If yes, how many **other pets** (please state what species)?___________________________________ n/a ⬜

***Visual Status***

What was your degree of **sight impairment/blindness**?

total ⬜

total with light perception/projection ⬜

partial w/ central field loss ⬜

partial w/ peripheral field loss ⬜

partial w/ no specific field loss ⬜

How much **useful vision** would you say you had ? none ⬜ a little ⬜ a lot ⬜

Did your **useful vision deteriorate** during the time you used this dog? yes ⬜ no or n/a ⬜

If yes, please state if vision loss was gradual or sudden, and if dog coped well or not: ____________ n/a ⬜

And the last question in this section:

If you could choose a **name** for this dog, what would it be? _____________________________________

*End of Section 2* (repeat as necessary for subsequent dogs)

**Section 3 - Guide Dog Rating**

This short section asks about your **most** and **least favourite qualities** regarding your guide dog/s.

Note: Choose which of the following questions to ask depending on multiple or single dog use.

*For Multiple dog use:*

Please name your **favourite** dog: ___________________________________________________________

What was the one feature or main quality that makes ‘this’ dog your favourite? ______________________

Please name your **least favourite** dog: ______________________________________________________

What was the one feature or main quality that made ‘this’ dog your least favourite? ___________________

*Or, if only one guide dog used:*

What is the one feature or main quality that you like **best** about this dog? __________________________

What is the one feature or main quality that you like **least** about this dog? __________________________

For each of the traits mentioned above, please categorise as either **work** or **non-work** related, and whether it is **behavioural** or **physical** in nature.

*End of section 3*

**Section 4 - Travel**

This section concerns various issues related to your **travel** (mobility) habits before you used a guide dog and when you use (or used) one. Some questions pertain to *independent travel*, which refers to your use of a mobility aid excluding a sighted guide. If any of the questions are not applicable to you, please let me know.

# *Non -visual Conditions*

I’ll begin by asking about any **non-visual conditions** you may have that restrict your mobility:

What, if any, non-visual conditions do you have that restrict or restricted your independent travel?

____________________________________________________________________ n/a ⬜

On a scale of 1-10, to what degree did these conditions **restrict** your independent travel **before** the use of a guide dog?

very little 1 2 3 4 5 6 7 8 9 10 extremely restricted n/a ⬜

On a scale of 1-10, to what degree do these conditions **restrict** your independent travel **when using** a guide dog?

very little 1 2 3 4 5 6 7 8 9 10 extremely restricted n/a ⬜

# *Mobility Aids Used*

The following questions pertain to your experiences with **mobility aids** in general.

**Before** you obtained a guide dog, which of the following mobility aids did you use?

long cane yes ⬜ no ⬜

electronic aid yes ⬜ no ⬜

low vision aid yes ⬜ no ⬜

sighted guide yes ⬜ no ⬜

no aid used yes ⬜ no ⬜

other yes ⬜ no ⬜

Which one did you use most often? ________________________________________________________

**After** you obtained a guide dog, which of the following mobility aids do you also use?

long cane yes ⬜ no ⬜

electronic aid yes ⬜ no ⬜

low vision aid yes ⬜ no ⬜

sighted guide yes ⬜ no ⬜

no aid used yes ⬜ no ⬜

other yes ⬜ no ⬜

guide dog yes ⬜ no ⬜

Which one do you use most often? ________________________________________________________

On a scale of 1-10, how *satisfied* were you with the amount and quality of **long cane O&M training** you received **before** you used a guide dog?

not at all 1 2 3 4 5 6 7 8 9 10 extremely satisfied n/a ⬜

# ***Travel Performance Indicators***

The next set of questions ask you to rate your independent **travel performance before** and **when using** a guide dog, depending on whether you considered your dog/s to be satisfactory or unsatisfactory mobility aids.

How many of your guide dog/s would you consider to be ‘unsatisfactory mobility aids’? _____ of _____.

*Where applicable:* As you have used more than one dog, please answer in as general a fashion as possible, by providing a single score for all your ‘good’ dogs and a single score for all your ‘bad’ dogs:

Note: As the questions progress, define orientation (O), mobility (M), and when used collectively, O&M, as three separate entities. Orientation refers to the ability to establish and maintain an awareness of one’s position in space relative to other objects in the environment, mobility refers to the act of purposeful movement using a tool such as a long cane, low vision aid, electronic aid or a guide dog, and O&M refers to the process of travelling through the environment safely and efficiently.

On a scale of 1-10, how would you rate your **O** performance **before** you used a guide dog?

very poor 1 2 3 4 5 6 7 8 9 10 excellent O performance

On a scale of 1-10, how would you rate your **O** performance **when using** a guide dog that you feel is satisfactory mobility aid?

very poor 1 2 3 4 5 6 7 8 9 10 excellent O performance n/a ⬜

On a scale of 1-10, how would you rate your **O** performance **when using** a guide dog that you feel is an unsatisfactory mobility aid?

very poor 1 2 3 4 5 6 7 8 9 10 excellent O performance n/a ⬜

On a scale of 1-10, how would you rate your **M** performance **before** you used a guide dog?

very poor 1 2 3 4 5 6 7 8 9 10 excellent M performance

On a scale of 1-10, how would you rate your **M** performance **when using** a guide dog that you feel is a satisfactory mobility aid?

very poor 1 2 3 4 5 6 7 8 9 10 excellent M performance n/a ⬜

On a scale of 1-10, how would you rate your **M** performance **when using** a guide dog that you feel is

an unsatisfactory mobility aid?

very poor 1 2 3 4 5 6 7 8 9 10 excellent M performance n/a ⬜

On a scale of 1-10, how would you rate your **O&M** performance collectively **before** you used a guide dog?

very poor 1 2 3 4 5 6 7 8 9 10 excellent O&M performance

On a scale of 1-10, how would you rate your **O&M** performance collectively **when using** a guide dog that you feel is a satisfactory mobility aid?

very poor 1 2 3 4 5 6 7 8 9 10 excellent O&M performance n/a ⬜

On a scale of 1-10, how would you rate your **O&M** performance collectively **when using** a guide dog that you feel is an unsatisfactory mobility aid?

very poor 1 2 3 4 5 6 7 8 9 10 excellent O&M performance n/a ⬜

On a scale of 1-10, how **difficult** was travelling in general **before** you used a guide dog?

not at all 1 2 3 4 5 6 7 8 9 10 extremely difficult

On a scale of 1-10, how **difficult** do you find travelling in general **when using** a guide dog that you feel is a satisfactory mobility aid?

not at all 1 2 3 4 5 6 7 8 9 10 extremely difficult n/a ⬜

On a scale of 1-10, how **difficult** do you find travelling in general **when using** a guide dog that you feel is an unsatisfactory mobility aid?

not at all 1 2 3 4 5 6 7 8 9 10 extremely difficult n/a ⬜

On a scale of 1-10, how **limited** was your travel in general **before** you used a guide dog?

not at all 1 2 3 4 5 6 7 8 9 10 extremely limited

On a scale of 1-10, how **limited** *is* your travel in general **when using** a guide dog that you feel is a satisfactory mobility aid?

not at all 1 2 3 4 5 6 7 8 9 10 extremely limited n/a ⬜

On a scale of 1-10, how **limited** is your travel in general **when using** a guide dog that you feel is an unsatisfactory mobility aid?

not at all 1 2 3 4 5 6 7 8 9 10 extremely limited n/a ⬜

On a scale of 1-10, how **often** did you travel **before** you used a guide dog?

not at all 1 2 3 4 5 6 7 8 9 10 very often

On a scale of 1-10, how **often** *do* you travel **when using** a guide dog that you feel is a satisfactory mobility aid?

not at all 1 2 3 4 5 6 7 8 9 10 very often n/a ⬜

On a scale of 1-10, how **often** *do* you travel **when using** a guide dog that you feel is an unsatisfactory mobility aid?

not at all 1 2 3 4 5 6 7 8 9 10 very often n/a ⬜

What is the main reason you travel more often or further, with a guide dog? _______________ n/a ⬜

***Journey Avoidance & Access*** (concerning environments, routes and destinations)

Please state any journeys you avoided **before** you used a guide dog? ________________________ n/a ⬜

Please state any journeys you avoid **when using** a guide dog? _____________________________ n/a ⬜

Please state any difficulties you had with access e.g. buildings/public transport **before** you used a guide dog. _______________________________________________________________________________ n/a ⬜

Please state any difficulties you have with access e.g. buildings/public transport **when using** a guide dog?

_______________________________________________________________________________ n/a ⬜

***Advantages & Disadvantages of Guide Dog Usage***

What are the **advantages** of guide dogs over other mobility aids? ________________________________

What are the **disadvantages** of guide dogs over other mobility aids? ____________________________

*End of Section 4*

Section 5 - Quality of Life

This brief section aims to explore the affect of using a guide dog on your **overall quality of life** including your health. Please feel free to comment as we go along.

*Where applicable:* As you have used more than one dog, please answer in a general fashion as possible by combining your overall experiences with guide dogs.

Did the use of a guide dog increase the number of **friendly approaches** by strangers? yes ⬜ no⬜

Comment: ____________________________________________________________________________

Did becoming a guide dog handler improve your level of **fitness**? yes ⬜ no ⬜

Comment: ____________________________________________________________________________

Did becoming a guide dog handler improve your **physical health**? yes ⬜ no ⬜

Comment: ____________________________________________________________________________

Did becoming a guide dog handler improve your **mental health**? yes ⬜ no ⬜

Comment: ____________________________________________________________________________

Did becoming a guide dog handler help you to **adjust** to your sight impairment or blindness? yes ⬜ no ⬜

Comment: ____________________________________________________________________________

Did becoming a guide dog handler improve your **quality of life** in general?

Comment: ____________________________________________________________________________

The next question concerns an issue regarding the end of the partnership. If you are currently working with your first dog, please feel free to project how you think you may feel at this eventuality.

Select option: actual feelings for previous dog/s ⬜ projected feelings for current dog ⬜

How does being ‘between’ (or without) a dog affect your quality of life? ___________________________

*End of section 5*

**Section 6 - Service Delivery & Miscellaneous**

This is the final section in the survey. To finish up, the following questions pertain to miscellaneous issues regarding guide dog use, and the services you receive from GDS. Again, please feel free to comment.

Do you envisage that you will use/**continue to use** a guide dog in the future? yes ⬜ no ⬜ don’t know ⬜

Comment: ____________________________________________________________________________

Have any of your guide dog/s worked as a guide for a **previous handler**? yes (specify) ⬜_______ no ⬜

Comment: ____________________________________________________________________________

­How long do you think a new guide dog be **preallocated**, if at all, to the handler, before guide dog training begins in order to facilitate bonding? _______________________________________________________

Comment: ____________________________________________________________________________

What is your preferred **location**, or locations, in which to **train** with a guide dog? ____________________

Comments: ____________________________________________________________________________

If given the choice, would you prefer to be the **legal owner** of your guide dog, as opposed to the dog

being the property of RNZFB Guide Dog Services? yes ⬜ no ⬜ don’t care ⬜

Comment: _____________________________________________________________________________

Did/do you think, your **expectations** change/will change from your first to subsequent dog/s? yes ⬜ no ⬜

Comment: _____________________________________________________________________________

If you had known then what you know now, would you have **returned** your first dog? yes ⬜ no ⬜ n/a ⬜

Comment: _____________________________________________________________________________

On a scale of 1-10, overall how **satisfied** are you with the services you have received from GDS to date?

not at all 1 2 3 4 5 6 7 8 9 10 completely satisfied

Is there anything you wish a guide dog could be **trained** to do in addition to the level of work you receive?

yes ⬜ no ⬜

Comment: _________________________________________________________________________ ____

How could the **‘matching process’** be improved? ______________________________________________

What else could GDS do to **improve its services** to you? ________________________________________

Well, that’s the end of the interview questions! Before we finish, are there any further comments or additions you would like to make? __________________________________________________________

*End of section 6*

# **Close of Interview**

*Thank you so much for participating in the survey. I hope you enjoyed the part you’ve played in helping to understand what makes a handler-guide dog team tick.*

*I’ll send you a summary of the results once the research is completed. If you have any questions about the study before then or wish to inform me about something, please email or phone me and I will get back to you as soon as possible.* [Give contact details if necessary.]

Notes: ________________________________________________________________________________

**End of Survey**
